# Mediating role of grip strength between depression symptoms and cognitive function: A cross-sectional study among older adults with diabetes in rural areas

**DOI:** 10.1371/journal.pone.0325442

**Published:** 2025-06-13

**Authors:** Xueyan Liu, Fangyun Luan, Lijuan Xiao, Yingjuan Cao

**Affiliations:** 1 School of Nursing and Rehabilitation, Cheeloo College of Medicine, Shandong University, Jinan, Shandong Province, P. R. China; 2 Department of Emergency, Qilu Hospital, Shandong University, Jinan, Shandong Province, P. R. China; 3 Department of Cardiology, Qilu Hospital, Shandong University, Jinan, Shandong Province, P. R. China; 4 Department of Nursing, Qilu Hospital, Shandong University, Jinan, Shandong Province, P. R. China; 5 Nursing Theory and Practice Innovation Research Center, Shandong University, Jinan, Shandong Province, P. R. China; University of Northumbria at Newcastle: Northumbria University, UNITED KINGDOM OF GREAT BRITAIN AND NORTHERN IRELAND

## Abstract

Older adults with diabetes are at an increased risk for both depression and cognitive decline. Depression symptoms have been linked to poorer cognitive function. Grip strength, an indicator of physical function, may play a role in this relationship. However, the specific mechanisms connecting depression, grip strength, and cognitive function in older adults with T2DM in rural areas are not fully understood. The objective of this study was to analyze the mediating role of grip strength in the relationship between depression symptoms and cognitive function. This Cross-sectional study was conducted from April to July 2023 in rural areas of China. To compare characteristics between male and female participants, one-way ANOVA and Chi-squared tests were used. Multivariate linear regression analysis was performed to examine the relationships between depression symptoms, grip strength, and cognitive function, adjusting for potential confounders. Bootstrap analysis, with 5000 resamples, was employed to assess the mediating role of grip strength in the relationship between depression symptoms and cognitive function. The study included 898 older adults with T2DM, with an average age of 69.73 years. Depression symptoms were found to be negatively associated with cognitive function. Grip strength was identified as a mediator in this relationship. Bootstrap analysis confirmed the partial mediating effect of grip strength, explaining 9% of the total effect of depression symptoms on cognitive function. These findings contribute to a better understanding of the underlying mechanisms between depression symptoms and cognitive function, elucidating the mediating role of grip strength in this association.

## Introduction

Diabetes is a common chronic metabolic disorder and has become a serious global public health problem. According to the International Diabetes Federation, in 2021, it is estimated that 537 million people worldwide have diabetes, which is expected to rise to 643 million by 2030 and to 783 million by 2045 [1]. In China, the number of people aged 20–79 with diabetes in China is 140.9 million in 2021, and it is expected to be 174.4 million by 2045 [1]. Type 2 diabetes mellitus (T2DM) accounts for approximately 90% of all diabetes cases and is predominantly prevalent in older adults [2].

Previous studies have revealed that people with T2DM are vulnerable to cognitive impairment in multiple domains [3,4], with a 60% increased risk of dementia [5]. Chronic hyperglycemia, insulin resistance, inflammation, and microvascular damage—common complications of T2DM—are known to negatively impact brain structure and function [6]. Insulin resistance, in particular, can impair brain function by promoting the aggregation of amyloid-beta protein and tau phosphorylation, resulting in hippocampal plasticity damage and triggering brain inflammatory responses [7]. Cognitive impairment may pose additional challenges for individuals with T2DM in adhering to complex treatment regimens, monitoring blood glucose levels, and making appropriate lifestyle choices [8]. Moreover, it may impair the ability to recognize and respond to symptoms of hypoglycemia or hyperglycemia, thereby increasing the risk of acute complications [9]. Although an increasing number of studies focus on the influencing factors of cognitive impairment, studies on this issue remain limited among T2DM patients [10]. It is widely recognized that the prevalence of cognitive impairment is higher in rural areas [11]. For instance, one study has shown that the prevalence of MCI among elderly T2DM patients in rural China is 50.22% [12]. Given these findings, investigating the risk factors for cognitive impairment in T2DM patients in rural areas is crucial for developing and implementing targeted prevention strategies.

The relationship between depression symptoms and T2DM has been widely explored [13]. Depression symptoms negatively impact blood glucose control in T2DM patients, increasing the risk of cardiovascular events and other complications, and worsening health outcomes [14]. Additionally, studies have indicated that depression symptoms are a risk factor for the onset and progression of cognitive impairment [15]. This means that the risk of cognitive impairment in people with T2DM is significantly increased by comorbid depression symptoms [16]. Grip strength is an essential indicator of the health status of middle-aged and older adults and is commonly used to assess overall muscle strength [17]. Low grip strength is a risk factor for early all-cause and cardiovascular mortality [18]. It is frequently observed in patients with T2DM [19]. Multiple studies have shown that older T2DM patients typically exhibit lower muscle mass index and muscle strength than non-diabetic individuals, with low grip strength being a typical feature [20,21]. A meta-analysis showed that for every 1 SD increase in grip strength, the risk of T2DM decreased by 13% [22]. The decline in skeletal muscle strength observed in individuals with T2DM is attributed to a combination of factors, including insulin resistance, direct damage induced by hyperglycemia, muscle fat infiltration, mitochondrial dysfunction, diabetic neuropathy, and physical inactivity [23,24]. Insulin resistance diminishes the sensitivity of muscle cells to insulin, leading to reduced glucose uptake and inadequate energy supply to the muscles, which subsequently hampers muscle growth and repair [25]. Advanced glycation end-products (AGEs) cross-link with proteins within muscle cells, thereby inhibiting muscle protein synthesis and potentially exacerbating the degradation of muscle proteins [26]. Previous studies have reported that low grip strength is associated with an increased risk of depression symptoms [27], and that strengthening grip strength may prevent or alleviate depression symptoms [28]. Furthermore, grip strength has been identified a valuable marker for assessing cognitive function and predicting cognitive impairment [29]. Older adults with weaker grip strength are also more vulnerable to multiple subtypes of dementia [30]. As a modifiable physiological indicator, grip strength offers a promising avenue for improving cognitive function, potentially serving as a target for intervention in the management of cognitive decline.

To the best of our knowledge, no studies have yet investigated the mediating role of grip strength in the relationship between depression symptoms and cognitive function among older adults with T2DM in rural areas of China. Therefore, this study aims to examine the mediating effect of grip strength, with the goal of further elucidating the underlying mechanisms between depression symptoms and cognitive function, and providing a theoretical basis for the development of targeted intervention strategies.

## Materials and methods

### Study design and sample

The study was conducted in rural areas of Binzhou City, Shandong Province, China, from April to July 2023. All methods adhered to relevant guidelines and regulations, including the Declaration of Helsinki. In this study, all participants provided informed consent. All procedures of the study complied with relevant ethical guidelines and were approved by the Institutional Ethics Committee (No. 2023-R-113). Before the study began, researchers explained the purpose, procedures, potential risks, and benefits of the study to the participants in detail and answered any questions they had. Due to the special circumstances of the participants (e.g., cultural or educational limitations), oral informed consent was obtained. All oral consents were obtained in the presence of the researchers and an independent witness (a community doctor) and were documented in the study records.

Cluster sampling was employed, using community health centers as the sampling units. All eligible individuals within each selected township were included as study subjects. Inclusion criteria were: (a) rural permanent residents; (b) ≥ 60 years; (c) diagnosed with T2DM. Exclusion criteria were: (a) history of mental or psychiatric illness; (b) severe neurological disorders that could potentially contribute to cognitive impairment, such as brain injuries, strokes, and dementia; (c) inability to complete the survey. Face-to-face interviews were conducted for all assessments. Finally, a total of 898 participants completed all questionnaires. Under the organization of community doctors, data were collected through face-to-face home visits with the elderly participants. Due to the fact that most of the participants were illiterate, trained researchers asked questions following a standardized script, and the participants provided their responses.

### Diagnosis of T2DM

We confirmed participants’ diabetes diagnosis through face-to-face interviews, using prior diagnostic certificates, records of antidiabetic medication use (including reasons for use), and medical records issued by healthcare institutions. An experienced internist verified all information to ensure diagnostic accuracy and reliability.

### Measurement

#### Depression symptoms.

We used the 15-item geriatric depression scale (GDS-15) to evaluate depression symptoms in older adults [31]. This scale contained 15 entries, “Yes” was scored as one point, and “No” was scored as zero points. Five items (Items 1, 5, 7, 11, 13) were reverse scored. Total scores ranged from 0 to 15, and a score of 6 or more points indicated the presence of depressive symptoms.

#### Grip strength.

Grip strength was measured with a spring-loaded hand-held grip strength meter. The participants were instructed on how to use a grip strength meter. They were seated in a chair with no armrests, holding a grip strength meter with their dominant hand, elbows flexed at 90°, and feet on the floor. Then, participants were asked to squeeze a grip strength meter with maximal effort. Three measurements were taken, and the maximum value was used as the result of the measurement [32].

#### Cognitive function.

This study used the Chinese version of the Montreal Cognitive Assessment-Basic (MoCA-B) to assess overall cognitive function. The scale provides a detailed assessment of cognitive function, which contained eight items addressing executive functions, language, orientation, calculation, abstract thinking, memory, visual perception, attention, and concentration [33]. Testing took approximately fifteen minutes, and the total score was thirty points. The MoCA- B scale was more suitable than the MoCA scale for people with low literacy.

#### Covariates.

We identified the following covariates as they may be related to cognitive function. Age, gender (Men, Women), marital status (Spouse survived, Widowed/Single), residence status (Living alone, Living with the spouse, Living with spouse and children, Living with or adjacent to children), education (Illiterate, 1–6, 7–9, ≥ 10), smoking status (Never or former smoking, Current smoking), drinking status (Never or former drinking, Current drinking), and hypertension (No, Yes). The above covariates were assessed through self-report by the participants. For hypertension, in addition to the self-reported medical history, we also conducted objective blood pressure measurements using a blood pressure monitor and simultaneously collected information on the use of antihypertensive medications.

### Statistical analysis

Analyses were conducted using SPSS software (version 26.0). Descriptive statistics were used for descriptive analysis, including means, standard deviations, frequencies, and percentages. Normality tests were conducted using the Kolmogorov-Smirnov test. One-way ANOVA and Chi-square tests were employed to compare differences in characteristics between male and female groups. Mediation analysis [34], following the approach outlined by Baron and Kenny (1986), was performed to estimate the mediation effect of grip strength on the association between depression symptoms and cognitive function. In the first step, multivariate linear regression was utilized to analyze the relationship between depression symptoms and cognitive function. In the second step, multivariate linear regression was employed to assess the relationship between depression symptoms and grip strength. Finally, multivariate linear regression was conducted to analyze the association between depression symptoms and cognitive function while including grip strength as a mediator. All relevant covariates were included in this analysis to control for potential confounding factors. To further validate the mediating effect of grip strength, a bootstrap analysis was performed. This method provided robust estimates of the mediation effect by generating confidence intervals through repeated sampling. A significance level of P < 0.05 was considered statistically significant for all analyses.

## Results

### Characteristics of participants

According to Table 1, 898 T2DM patients were enrolled in this study, comprising 322 men and 576 women. The average age of participants was 69.73 years (SD = 6.06). Of the participants, 383 (42.70%) were illiterate. Women exhibited significantly lower levels of education compared to men, with an illiteracy rate of 42.7%. Women also exhibited a higher prevalence of hypertension, with 453 women (78.60%) affected, compared to men. Additionally, women had lower grip strength (mean = 15.68 ± 5.00), more pronounced depressive symptoms (mean = 5.26 ± 2.41), and poorer cognitive function (mean = 13.16 ± 5.02). In contrast, men were more likely to report smoking and alcohol consumption. Despite these differences, no significant variations were observed between men and women in terms of age, marital status, and hypertension prevalence.

**Table 1:** Characteristics of the older adults with type 2 diabetes in rural China by gender.

Characteristics	Total (N = 898) n (%)	Men (N = 322) n (%)	Women (N = 576) n (%)	P-value
Age (years), mean (SD)	69.73 (6.06)	69.79 (6.13)	69.69 (6.02)	0.82
Marital status				0.311
Spouse survived	697 (77.60)	256 (79.50)	441 (76.60)	
Widowed/Single	201 (22.40)	66 (20.50)	135 (23.40)	
Residence status				< 0.001
Living alone	131 (14.60)	57 (17.70)	74 (12.80)	
Living with spouse	552 (61.50)	212 (65.80)	340 (59.50)	
Live with spouse and children	127 (14.10)	36 (11.20)	91 (15.80)	
Living with or adjacent to children	88 (9.80)	17 (5.30)	71 (12.30)	
Education (years)				< 0.001
Illiterate	383 (42.70)	40 (12.40)	343 (42.70)	
1-6	274 (30.50)	108 (33.50)	166 (30.50)	
7-9	157 (17.50)	109 (33.90)	48 (17.50)	
≥10	84 (9.40)	65 (20.20)	19 (9.40)	
Smoking status				< 0.001
Never or former smoking	764 (85.10)	228 (70.80)	536 (93.10)	
Current smoking	134 (14.90)	94 (29.20)	40 (6.90)	
Drinking status				< 0.001
Never or former drinking	763 (85.00)	205 (63.70)	558 (96.90)	
Current drinking	135 (15.00)	117 (36.30)	18 (3.10)	
Hypertension				0.069
No	209 (23.3)	86 (26.70)	123 (21.40)	
Yes	689 (76.70)	236 (73.30)	453 (78.60)	
Grip strength, mean (SD)	18.24 (6.68)	22.84 (6.84)	15.68 (5.00)	< 0.001
GDS-15, mean (SD)	4.96 (2.39)	4.43 (2.26)	5.26 (2.41)	< 0.001
MoCA, mean (SD)	14.49 (5.36)	16.86 (5.14)	13.16 (5.02)	< 0.001

### Mediating effect of grip strength on the relationship between depression symptoms and cognitive function

**Fig 1:**
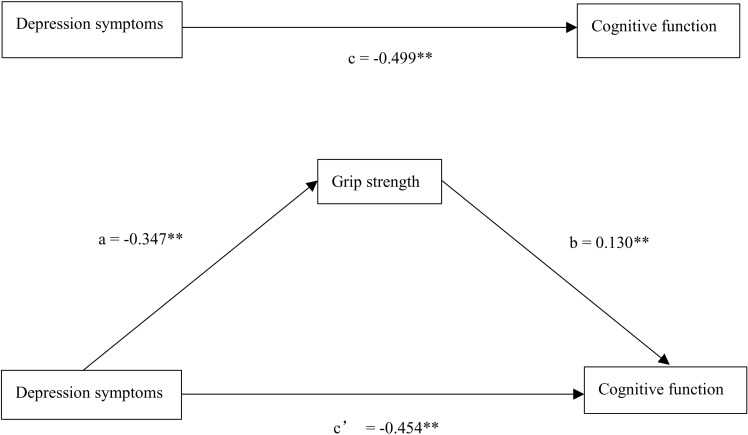
Mediating effect diagram. (**P < 0.001).

Table 2 shows the mediation analysis results. We examined the mediating role of grip strength on the relationship between depression symptoms and cognitive function in three steps. In Model 1, controlling for age, gender, marital status, residence status, education, smoking status, drinking status, hypertension, depression symptoms were significantly associated with cognitive function (*β* = −0.222, *P* < 0.001). In Model 2, age, gender, marital status, residence status, education, smoking status, drinking status, hypertension, depression symptoms showed a significant relationship with grip strength (*β* = −0.124, *P* < 0.001). In Model 3, controlling for age, gender, marital status, residence status, education, smoking status, drinking status, hypertension, both depression symptoms (*β* = −0.202, *P* < 0.001) and grip strength (*β* = 0.163, *P* < 0.001) showed a significant relation with cognitive function. The results of the bootstrap mediation test are presented in Table 3. There was a significant indirect effect of depression symptoms via grip strength (95% *CI* = −0.076, −0.020), and depression symptoms had an impact of −0.045 that was produced by grip strength as a mediator on cognitive function. The mediating effect of grip strength can explain 9% of the total effect of depression symptoms on cognitive function. Fig 1 shows the mediating model.

**Table 2:** The mediating effect of grip strength between depression symptoms and cognitive function.

Model 1	Depression symptoms Cognitive function				P
Characteristics	B	SE	β	t	
Age (years), mean (SD)	−0.138	0.025	−0.156	−5.412	< 0.001
Gender	−1.143	0.398	−0.102	−2.875	0.004
Marital status	−0.367	0.368	−0.029	−0.997	0.319
Residence status	0.075	0.185	0.011	0.405	0.685
Education (years)	1.856	0.183	0.341	8.148	< 0.001
Smoking status	−0.620	0.448	−0.041	−1.383	0.167
Drinking status	1.014	0.475	0.068	2.134	0.033
Hypertension	0.216	0.351	0.107	0.617	0.537
Depression symptoms	−0.499	0.065	−0.222	−7.655	<0.001
**Model 2**	**Depression symptoms** **Grip strength**				
Characteristics	B	SE	β	t	P
Age (years), mean (SD)	−0.144	0.032	−0.131	−4.518	< 0.001
Gender	−5.686	0.499	−0.409	−9.404	< 0.001
Marital status	−0.389	0.462	−0.024	−0.842	0.400
Residence status	−0.059	0.232	−0.007	−0.254	0.800
Education (years)	0.645	0.229	0.095	2.814	0.005
Smoking status	−0.494	0.562	−0.026	−0.879	0.380
Drinking status	1.747	0.596	0.094	2.931	0.003
Hypertension	−0.346	0.440	−0.022	−0.787	0.431
Depression symptoms	−0.347	0.082	−0.124	−4.239	<0.001
**Model 3**	**Depression symptoms, Grip strength** **Cognitive function**				
Characteristics	B	SE	β	t	P
Age (years), mean (SD)	−0.119	0.025	−0.135	−4.679	<0.001
Gender	−0.399	0.420	−0.036	−0.950	0.342
Marital status	−0.317	0.364	−0.025	−0.870	0.384
Residence status	0.083	0.183	0.012	0.453	0.651
Education (years)	1.771	0.181	0.326	9.771	< 0.001
Smoking status	−0.555	0.443	−0.037	−1.254	0.210
Drinking status	0.786	0.472	0.052	1.667	0.096
Hypertension	0.262	0.346	0.021	0.756	0.450
Depression symptoms	−0.454	0.065	−0.202	−6.981	<0.001
Grip strength	0.131	0.026	0.163	4.951	<0.001

**Table 3:** Mediating model examination by bootstrap test.

	Depression symptoms Cognitive function			
	Effect	SE	LL 95%CI	UL 95%CI
Total effect	−0.499	0.065	−0.627	−0.371
Direct effect	−0.454	0.065	−0.581	−0.326
Indirect effect	−0.045	0.014	−0.076	−0.020

## Discussion

This study explored the role of grip strength in the relationship between depression symptoms and cognitive function among older adults with T2DM in rural areas. The findings reveal a significant association between depression symptoms and cognitive function, with grip strength playing a mediating role in this relationship. Specifically, more severe depression symptoms were linked to poorer cognitive performance, and this association was partly explained by lower grip strength. Our findings suggest that improving grip strength could serve as an effective strategy to mitigate cognitive decline associated with depression, especially in older adults with T2DM in rural settings. By focusing on both depression symptoms and physical strength, healthcare strategies could help prevent or slow down cognitive decline, thus improving overall quality of life for this vulnerable population.

Depression symptoms are prevalent in older adults with T2DM and contribute to the increased burden of diabetes and its adverse effects on glycemic control. Our study underscores the importance of addressing depression in this population. We found a clear link between depression symptoms and cognitive function, where more severe depression symptoms were associated with poorer cognitive performance. This association has been documented in prior research [35,36], emphasizing the need to better understand the mechanisms underlying this relationship in order to develop effective therapeutic interventions. However, the mechanisms by which depression affects cognitive function remain complex and not fully understood. Chronic psychological stress, commonly seen in individuals with depression, can lead to hypercortisolism, which inhibits hippocampal neurogenesis and may result in hippocampal atrophy [37]. Depression symptoms may reduce daily activities and physical exercise, which can affect cerebral blood flow and neuroplasticity, thereby accelerating cognitive decline [38]. This suggests that depression could contribute to cognitive impairment through both neurobiological and behavioral pathways, such as changes in brain structure and function, as well as reduced physical activity.

In this study, we also observed a significant relationship between grip strength and cognitive function, consistent with findings from previous studies [29,39]. A recent meta-analysis demonstrated that lower grip strength is strongly associated with cognitive decline and dementia [40]. Grip strength is often considered an indicator of overall physical health and muscle function. Lower levels of inflammatory markers such as C-reactive protein, IL-6, and TNF-α have been shown to correlate with reduced muscle strength and mass [41,42]. Inflammation is widely recognized as a critical factor in the pathogenesis and progression of cognitive impairment [43]. Furthermore, lower muscle mass is linked to insulin resistance, which has been shown to increase the risk of cognitive impairment [44].

Studies have shown that lower grip strength is often associated with greater severity of depression symptoms [27,45]. Some research suggests that chronic conditions, such as T2DM, can impact both depression and grip strength, which further complicates their relationship [46]. Despite the growing body of literature, studies specifically examining the link between grip strength and depression in individuals with T2DM remain limited. Our study fills this gap by revealing a negative correlation between depression symptoms and grip strength in older adults with T2DM. The association between depression symptoms and reduced grip strength may be particularly pronounced in T2DM patients, as diabetes itself can impair muscle function. Depression symptoms are known to lead to hormonal imbalances or changes in neurotransmitter levels, which can negatively impact muscle strength [47]. Moreover, individuals with depression symptoms often have reduced motivation and physical activity, which can further contribute to the decline in grip strength [48].

Our study found that grip strength mediates the relationship between depression symptoms and cognitive function. In other words, depression symptoms indirectly affect cognitive function by weakening grip strength. This finding suggests that improving grip strength could be an effective way to reduce cognitive decline caused by depression. It emphasizes the importance of including strength training as a key component in the comprehensive care plan for elderly patients with T2DM. However, it is important to note that only 9% of the relationship between depression and cognitive function could be explained by grip strength. This indicates that other factors also contribute to the observed relationship.

This study has several limitations. First, due to the cross-sectional design, it is not possible to establish causal relationships between depression symptoms, grip strength, and cognitive function. Longitudinal studies are needed to determine the directionality and causality of these associations. Second, individuals with severe cognitive impairment were excluded from the study because they were unable to complete the assessments. This exclusion may have led to an underestimation of the true extent of cognitive impairment among older adults with T2DM, potentially biasing the results. Third, participants were recruited from a single region, which limits the generalizability of the findings to older adults with T2DM in rural China. Future studies should include a more diverse and representative sample to enhance the external validity of the results. Additionally, although the MoCA-B scale is particularly suitable for individuals with low literacy levels, some participants with very low educational backgrounds may still experience difficulties understanding certain items, which could affect the accuracy of the assessment results. Finally, this study did not control for all potential confounding factors, such as glycemic control and nutrition status.

## Conclusions

To the best of our knowledge, this was the first study to examine the mediating role of grip strength on the relationship between depression symptoms and cognitive function among older adults with T2DM in rural areas. In the current study, we found that grip strength has a partially mediating effect on this relationship. This suggested that older adults with T2DM should be encouraged to enhance overall body muscular strength to improve cognitive function and maintain mental health.

## Relevance to clinical practice

Regular screening for depression symptoms in older adults with T2DM should be a priority. Early identification and treatment of depression can help mitigate its negative impact on cognitive function. Healthcare providers should incorporate routine mental health screenings alongside physical health assessments to ensure timely intervention.

Implementing structured physical exercise programs designed specifically for older adults with T2DM, such as resistance training or aerobic exercises, can significantly benefit both physical and cognitive health. Resistance training, for example, is known to improve grip strength and overall physical function, while also contributing to enhanced cognitive performance. These interventions should be a core component of comprehensive care strategies. Additionally, other forms of physical exercises, including aerobic and balance exercises, could complement strength training in reducing cognitive decline and improving overall brain health. Healthcare providers in rural areas should be trained to recognize the interplay between physical and mental health and to offer comprehensive care plans that integrate both. Rural healthcare professionals may face challenges such as limited access to specialized care and insufficient training in mental health. Solutions may include telemedicine programs that offer virtual consultations and mental health screenings, as well as mobile health units that bring services directly to rural communities. These resources can bridge the gap in access to healthcare services in areas where medical facilities are limited.

Moreover, leveraging local community resources is key to supporting both mental and physical health interventions. Local health centers and community groups can play a crucial role in delivering mental health screenings, physical exercises, and educational programs. Empowering community health workers to deliver these services ensures that rural populations receive the care and support they need. Policymakers should prioritize the development and allocation of resources for healthcare initiatives tailored to the specific needs of rural populations. Increased funding for rural health centers, specialized training for healthcare providers, and support for community-based health programs are critical. Investing in such programs will lead to significant improvements in the well-being of older adults with T2DM, enabling them to better manage both their mental and physical health.
